# A trick for visualisation of the radial tuberosity in a biceps tendon repair

**DOI:** 10.1308/003588412X13373405387096l

**Published:** 2012-05

**Authors:** NA Smith, R Jordan, D Wainwright

**Affiliations:** University Hospitals Coventry and Warwickshire NHS Trust,UK

**Figure 1:**
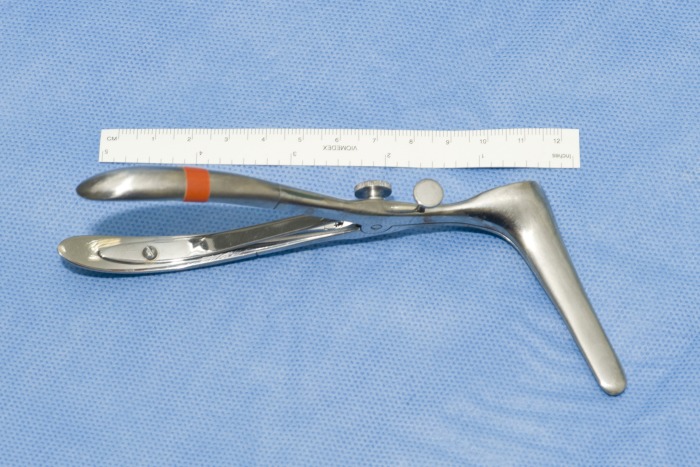
Killian nasal speculum

**Figure 2:**
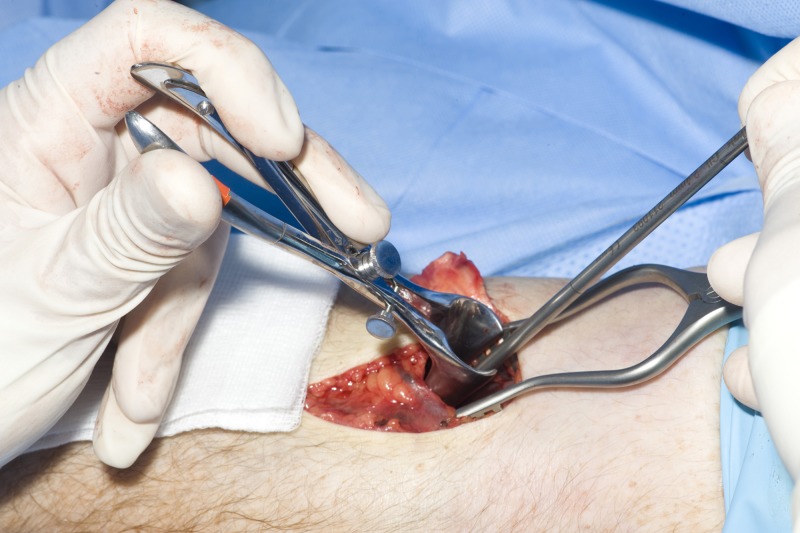
A Killian nasal speculum allows excellent visualisation of the radial tuberosity while avoiding damage to surrounding structures.

The modified Henry approach to a biceps tendon repair allows a direct approach to the radial tuberosity although clear visualisation of it from this approach is difficult. It is particularly important to visualise the radial tuberosity well in order to avoid poor placement of the bone anchors, with the potential to give poor function of the biceps tendon. We have found using a Killian nasal speculum (Fig 1) allows excellent visualisation of the radial tuberosity while avoiding damage to surrounding structures (Fig 2). The radial tuberosity is directly visualised, minimising any risk of incorrect placement of drill holes for the anchor sutures.

